# Prophylactic salpingo-oophorectomy in *BRCA1* mutation carriers and postoperative incidence of peritoneal and breast cancers

**DOI:** 10.1186/s13048-016-0220-4

**Published:** 2016-02-29

**Authors:** Janusz Menkiszak, Anita Chudecka-Głaz, Jacek Gronwald, Aneta Cymbaluk-Płoska, Aleksander Celewicz, Maria Świniarska, Małgorzata Wężowska, Ryszard Bedner, Dorota Zielińska, Paulina Tarnowska, Jerzy Jakubowicz, Zbigniew Kojs

**Affiliations:** Department of Gynecological Surgery and Gynecological Oncology of Adults and Adolescents, Pomeranian Medical University, SPSK-2, 70-111 Szczecin Al. Powstańców Wielkopolskich 72, Szczecin, Poland; Department of Genetics and Pathology; International Hereditary Cancer Center, Pomeranian Medical University, Al. Powstańców Wielkopolskich 72, 70-111 Szczecin, Poland; Radiation Oncology Department, Centre of Oncology, Maria Sklodowska-Curie Memorial Institute Cracow Branch, Garncarska 11, 31-115 Kraków, Poland; Department of Gynecologic Oncology, Centre of Oncology, Maria Sklodowska-Curie Memorial Institute, Cracow Branch, Garncarska 11, 31-115 Kraków, Poland; Department of the Clinical Oncology the West Pomeranian Centre of the Oncology in Szczecin, ul. Strzałowska 22, 71-730 Szczecin, Poland

**Keywords:** Prophylactic bilateral salpingo-oophorectomy, Risk-reducing salpingo-oophorectomy, *BRCA1*, Peritoneal cancer, Breast cancer

## Abstract

**Background:**

There are no effective methods of diagnosis of early-stage ovarian cancer. Conservative care over patients at high risk of ovarian and breast cancers is ineffective. Prophylactic surgery is considered the best prophylaxis among BRCA1/BRCA2 carriers.

**Methods:**

One hundred ninety-five patients, carriers of one of three most common mutations of the BRCA1 gene (Am J Hum Genet: 66: (6)1963-1968, 2000) in the Polish population (5382insC, 4153delA and C61G), who undergone prophylactic salpingo-oophorectomy. The study group consisted of consecutive mutation carriers living in Poland, in the West Pomeranian province. Histopathological examination of the surgical material failed to reveal presence of malignancy.

**Results:**

During follow-up we diagnosed two peritoneal cancers and 14 breast cancers. Diagnosis of breast cancer before prophylactic surgery increased the risk of peritoneal cancer almost three times. Time from diagnosis of breast cancer to prophylactic surgery increased the risk of peritoneal cancer after prophylactic surgery. This was strongly expressed (HR = 5.0; *p =* 0.030) in cases of over five-year-long delay in prophylactic surgery. Diagnosis of breast cancer before prophylactic surgery correlated with the risk of death (*p =* 0.00010). Presence of 5382insC mutation decreased and C61G mutation increased the risk of peritoneal cancer (*p =* 0.049 vs. *p =* 0.013).

**Conclusions:**

Occurrence of primary peritoneal cancer after prophylactic surgery is similar to that reported in international literature. Primary breast cancer occurred less often than in international literature. We suspect that the risk of development of breast cancer among BRCA1 carriers undergoing prophylactic surgery can differ in a population. The next goal should be to study the molecular basis for the risk of development of malignancies in any population. Carriers of BRCA1 gene diagnosed with breast cancer should undergo prophylactic surgery within five years from the diagnosis of breast cancer.

## Background

At the current state of knowledge there are no effective methods enabling identification of ovarian cancer at an early stage [1–5]. Conservative methods of prevention also failed to reduce the mortality due to ovarian cancer in a group of high-risk patients – carriers of the *BRCA1* or *BRCA2* gene mutations [6–13]. Other methods of conservative care over high-risk of ovarian and breast cancer patients(*BRCA1* or *BRCA2* carriers) have proven ineffective [1, 11, 14, 15].

According to international literature prophylactic surgery, referred to as PBSO (Prophylactic Bilateral Salpingo-Oophorectomy), RRSO (Risk-Reducing Salpingo-Oophorectomy), RRBSO (risk-reducing bilateral salpingo-oophorectomy) or BSO, is currently considered the best prophylaxis among *BRCA1/BRCA2* mutation carriers [1, 16–20].

In *BRCA1/BRCA2* mutation carriers, PBSO/RRSO not only decreases the risk of development of ovarian cancer by 80-90 % and breast cancer by 40–50 %, but also reduces mortality due to cancer of the genital tract and overall mortality [21–24].

According to Kotsopoulos et al., salpingo-oophorectomy is equally effective in prevention of breast cancer in women after natural menopause (*p =* 0.006) [25]. Moreover, prophylactic salpingo-oophorectomy protects patients with breast cancer who are also *BRCA1/BRCA2* carriers from developing ovarian cancer. Metcalfe et al. demonstrated that 25 % of deaths in this group of patients, particularly with a diagnosis of stage 1 breast cancer, is caused by ovarian cancer. The fact that in this group of patients systemic treatment did not significantly influence the risk of development of ovarian cancer [26] is also an argument in favor of prophylactic surgery.

In this work we present the results of our observations in patients with mutation in *BRCA1* gene after prophylactic genital tract surgery in relation to development of peritoneal or breast cancers. We assessed selected risk factors for peritoneal cancer and compared chosen characteristics depending on the presence or absence of breast cancer diagnosis in a group of patients before prophylactic surgery.

Goal:

1. To assess the incidence of peritoneal and breast cancer among carriers of the *BRCA1* gene mutation after prophylactic salpingo-oophorectomy.
2. To analyze selected risk factors for peritoneal cancer in patients after prophylactic surgery.
3. To assess selected characteristics among patients subjected to surgery depending on the diagnosis of breast cancer before prophylactic operation.

## Methods

The material consisted of 195 patients, carriers of one of three mutations in *BRCA1* gene most commonly occurring in the Polish population (*5382insC, 4153delA and C61G*) [27], subjected to prophylactic salpingo-oophorectomy. Patients underwent prophylactic surgery over a period from 15 Sept 1999 to 31 Dec 2010. Study included consecutive mutation carriers from the West Pomeranian province, Poland, treated with surgery at the Department and Clinic of Surgical Gynecology and Gynecological Oncology (adults and children) of the Pomeranian Medical University in Szczecin, who were not diagnosed with malignancy in histopathological examination of excised material. Median age of patients at the time of surgery was 47 years (31–78 years). Median follow-up time was 80 months (4–153 months).

As much as 41.03 % (80/195) of patients were treated for breast cancer before prophylactic surgery. Patients who had been treated for breast cancer before prophylactic surgery were undergoing prophylactic salpingo-oophorectomy at an older age than patients without the diagnosis of breast cancer before surgery. Both patients diagnosed with primary peritoneal cancer after prophylactic surgery, aside from salpingo-oophorectomy, had undergone hysterectomy. Proportions of specific *BRCA1* mutations were the following: 65.64 % (128/195) of patients were *5382insC* carriers, 24, 62 % (48/195) had *C61G* mutation, and 9.74 % (19/195) subjects had mutation of the *4153 DelA* type. As much as 6.67 % (13/195) of patients died during follow-up.

### Statistical analysis

All continuous variables were checked for normal distribution with Kolmogorov-Smirnov test. Such variables were described as means, standard deviations, medians, quartiles, as well as minimal and maximal values. Statistical differences between two groups were checked using Student’s *t* test or Mann-Whitney’s test. Non-continuous variables were described through absolute numbers and frequency of occurrence. Pearson’s *χ*^2^ test or exact Fisher’s test were used to assess the relationship between non-continuous variables.

A logistic regression model was used in order to assess the risk of occurrence of a pathology depending on the presence of various factors. Results were described by relative risk (OR) with 95 % confidence interval and a *p*-value indicating statistical significance. In this model, probability was assessed using Pearson’s *χ*^2^ test or Fisher’s exact two-tailed test.

Differences were considered statistically significant in all conducted tests with *p <* 0.05. Level of statistical significance *p =* 0.051-0.099 was considered a trend with borderline statistical significance.

Statistical analyses were performed using STATA 11 software (license no. 30110532736). The bioethics committee of the Pomeranian Medical University approved (ref. no. BN-001/202/03) for the research to be carried out.

## Results

During the follow-up time (median time was 80 months) there were 2 (2/195) cases of primary peritoneal cancer, 14 (14/195) cases of breast cancer, including 9 (9/195) primary cancers (Table 1), diagnosed in our group of *BRCA1* mutation carriers subjected to prophylactic salpingo-oophorectomy.

**Table 1:** Number of cancers and type of organ affected by cancer in patients followed up after prophylactic surgery

		No.				
		Number %/(n)	All primary breast cancers %/(n)	All breast cancers %/(n)	All primary peritoneal and breast cancers %/(n)	Total %/(n)
Organ						
Peritoneum		1.03 % (2/195)	-	-	5.64 % (11/195)	8.21 % (16/195)
Breast	Primary	3.08 %(6/195)	4.63 %(9/195)	7.18 %(14/195)		
	II – primary	1.54 % (3/195)				
	Recurrence	2.56 % (5/195)	N/A		N/A	

All breast cancers as well as primary breast cancers were diagnosed in our material more frequently than peritoneal cancer and that difference was statistically significant, with *p =* 0.0022 (OR = 7.46; 95 % CI: 1.67–68.29) vs. *p =* 0.0323 (OR = 4.67; 95 % CI: 1.10–44.10). Primary breast cancer was 2.27 times more frequent than breast cancer recurrence, but that relationship was not statistically significant (OR = 2.27; 95 % CI: 0.71–8.49).

Time of cancer diagnosis after prophylactic surgery was significantly shorter among patients with *5382insC* mutation compared with *C61G* and *4153DelA* mutations combined, *p =* 0.0271 (median: 23 vs. 62 months).

Diagnosis of breast cancer in a patient before prophylactic surgery increased the risk of peritoneal cancer almost three times, but this characteristic was on the border of statistical significance (*p =* 0.088). Carrying a *5382insC* mutation decreased the probability of peritoneal cancer, while *C61G* mutation increased such likelihood, *p =* 0.049 and *p =* 0.013, respectively. Among carriers of *4153DelA* mutation the risk of development of peritoneal cancer was neither increased nor decreased; that characteristic was not statistically significant (Table 2).

**Table 2:** Risk of occurrence of peritoneal cancer in the whole group of patients (*n =* 195) depending on the characteristics assessed with Cox regression model

	Data			
	Risk factors	HR	95 % CI	p
Dependent variable				
Peritoneal cancer	Breast cancer before surgery	2.95	0.75 175.43	0.088
	Age at time of surgery (years)	1.15	0.96 1.37	0.125
	*5382insC* mutation	0.25	0.00 0.99	0.049
	*C61G* mutation	6.39	1.62 379.51	0.013
	*4153DelA* mutation	0.90	0.07 18.50	0.641

Time from the diagnosis of breast cancer in a patient before prophylactic surgery to the procedure itself was a feature increasing the risk of peritoneal cancer in a significant manner. That characteristic was particularly strong (HR = 5.0; *p =* 0.030) in cases where the decision of prophylactic surgery was delayed by more than five years (Table 3).

**Table 3:** Risk of occurrence of peritoneal cancer in the group of patients with diagnosed breast cancer before prophylactic surgery (*n =* 80) depending on the characteristics assessed with Cox regression model

	Data				
	Characteristic	Risk factor	HR	CI 95 %	*p*
Dependent variable					
Peritoneal cancer	Time from diagnosis of breast cancer prior to prophylactic surgery to prophylactic surgery	Years	1.14	1.00 1.29	0.043
	Time from diagnosis of breast cancer prior to prophylactic surgery to prophylactic surgery	Years > =5 vs. <5	5.00	1.23 300.17	0.030
	Age at the moment of breast cancer diagnosis in a patient before prophylactic surgery	Years	0.93	0.75 1.14	0.488

Among all features selected for analysis among *BRCA1* carriers diagnosed with breast cancer before prophylactic surgery, only the survival feature was statistically significant (*p =* 0.00010). Patients diagnosed with cancer before prophylactic surgery died more frequently. In that group of patients increased probability of development of any cancer or peritoneal cancer was on the border of statistical significance. Remaining features subjected to analysis, including type of mutation, were not statistically significant (Table 4).

**Table 4:** Evaluation of selected characteristics among patients depending on the diagnosis of breast cancer before prophylactic surgery

Characteristic		Breast cancer before surgery					
		Not identified *n =* 115		Identified *n =* 80		N	p
Death		1	0.87 %	12	15.00 %	13	*p =* 0.00010
Development of cancer		6	5.22 %	10	12.50 %	16	*p =* 0.06835
Development of peritoneal cancer		0	0.00 %	2	2.50 %	2	*p =* 0.08832
Development of breast cancer		6	5.22 %	8	10.00 %	14	*p =* 0.20318
Development of primary breast cancer		6	5.22 %	3	3.75 %	9	*p =* 0.63096
	*5382insC*	78	67.83 %	50	62.50 %	128	*p =* 0.44111
Type of mutation	*C61G*	24	20.87 %	24	30.00 %	48	*p =* 0.14543
	*4153DelA*	13	11.30 %	6	7.50 %	19	*p =* 0.37823

## Discussion

In 1982 Jeanne Tobacman et al. described the first three cases of peritoneal cancer after prophylactic oophorectomy among 28 patients from families at high risk of ovarian cancer [28]. Many studies have been published since then. Sitzmann and Wiebke [29] point to several important facts in one of the most recent metaanalyses evaluating, among other things, the frequency of occurrence of peritoneal cancer after salpingo-oophorectomy in patients with *BRCA1/BRCA2* mutation. Firstly, a great majority of authors report the number of diagnosed peritoneal cancers at a level of 0.8–1.8 % [22, 30–36].

Casey et al. diagnosed 5 cases of peritoneal cancer among 118 carriers of *BRCA1* mutation. In one case the patient had undergone oviduct-sparing surgery [37]. Among three cases of peritoneal cancer diagnosed in *BRCA1* carriers after PBSO, Mæhle et al. associated that fact with presence of ovarian tissue left behind after previous surgeries [38]. Oliver et al. diagnosed 11.5 % (3/26 – *BRCA1* mutation) of peritoneal cancers after prophylactic surgery, but the study group consisted of patients after oviduct-sparing surgery. In the second group of patients subjected to salpingo-oophorectomy the same authors did not find any cases of peritoneal cancer during the follow-up period (0/58 – *BRCA1*). However, researchers admit that it might have been due to very short mean follow-up time, which amounted to 12 months [39]. Likewise, after 16 months of follow-up, Gaarenstroom et al. did not diagnose peritoneal cancer in any of 114 patients (57 patients were carriers of *BRCA1* mutation) following prophylactic salpingo-oophorectomy [40].

Laki et al. did not find any cases of peritoneal cancer among their patients following mean follow-up time of 40 months either [41]. Similarly, there were no cases of primary peritoneal cancer in an 8.17-year follow-up in a study by Evans et al. that included *BRCA1/BRCA2* mutation carriers (160 patients, including 104 subjects with *BRCA1* mutation) after salpingo-oophorectomy [42].

Authors who failed to find cases of peritoneal cancer after prophylactic salpingo-oophorectomy most often explain it by short follow-up time. Such an argument is no longer valid for the analysis of work by Evans et al. Small size of the group is also often given as a reason for the lack of such a finding [42].

In our analysis we acquired a result almost identical with regard to the frequency of diagnosis of peritoneal cancer among patients after salpingo-oophorectomy to that obtained by Rhiem et al. – 1.03 % vs. 1.09 % (2/195 vs. 1/92 – *BRCA1* gene mutations). The time of the first diagnosis of peritoneal cancer was also similar – in our material it amounted to 30 months, while according to Rhiem et al. – 26 months [34]. In our study the second peritoneal cancer was diagnosed after 62 months.

The second, very important conclusion ensuing from this research is such that after prophylactic surgery peritoneal cancer develops more frequently in *BRCA1* mutation carriers. Peritoneal cancer was very rare among patients with *BRCA2* mutation. In their metaanalysis, Sitzmann and Wiebke [29] presented results of 12 research teams and in only one study by Finsch et al. peritoneal cancer was found in a patient with a mutation in the *BRCA2* gene [33].

However, it should be emphasized, that while there are thousands of examined patients and mean follow-up time from prophylactic surgery to the diagnosis of peritoneal cancer exceeds 6 years, the proportion of mutations in both *BRCA* genes found in patients with primary peritoneal cancer diagnosed after salpingo-oophorectomy has evidently changed – 28 *BRCA1* vs. *BRCA2* [21].

Due to a small number of subjects after prophylactic surgery with *BRCA2* gene mutation, we did not take their data into consideration.

In our material we diagnosed 14 cases (14/195 = 7.18 %) of breast cancer, including 6 cases in patients without the diagnosis of breast cancer before prophylactic surgery, 3 cases of second primary breast cancer and 5 cases of breast cancer recurrence. Thus, primary breast cancers identified during the follow-up time constituted only 4.63 % (9/195) of all cases (Table 1).

Results obtained for primary breast cancers in our material were somewhat smaller than values reported by Casey et al. – 4.63 % vs. 5.93 % (7/118 – *BRCA1* mutation) over a shorter median follow-up time – 6.67 vs. 8.3 years, respectively [37].

Similar overall results were obtained by Kauff et al.– 7.89 % (15/190 – *BRCA1* mutation), with mean follow-up time shorter almost by half [32], and Powell et al. – 6.35 % (4/63) among *BRCA1* carriers, with median observation time almost 1/3 shorter compared to our study. Moreover, in a detailed analysis researchers emphasize that most of these cases were breast cancer recurrences [36]. Domchek et al. acquired significantly higher results with respect to breast cancer diagnoses among *BRCA1* mutation carriers subjected to prophylactic salpingo-oophorectomy over an almost 5-year-long mean follow-up time – 13.6 % (51/374 – *BRCA1).* Moreover, quantitative values were comparable regardless of a diagnosis of breast cancer before prophylactic surgery [22].

Similarly high values were obtained by Fakkert et al. – 11.54 % (12/104 – *BRCA1*) over a median 3-year observation time [43] and Laki et al. – 10.71 % (6/56 – *BRCA1*) [41].

Ramon Y Cajal et al. presented very high quantitative values – researchers diagnosed as many as 5 cases of breast cancer among 25 carriers of *BRCA1* gene mutation (20 %–5/25) subjected to prophylactic salpingo-oophorectomy. In that case median follow-up time was 49 months [44].

Likewise, a very high (over 20 %) proportion of breast cancer diagnoses among BRCA1 mutation carriers after prophylactic bilateral oophorectomy was obtained by Shah et al. with a median follow-up time from surgery to the diagnosis of cancer amounting to 3.6 years [45].

Available literature is in agreement with regard to higher incidence of breast cancer than peritoneal cancer among *BRCA1* carriers undergoing prophylactic salpingo-oophorectomy [22, 32, 36, 41, 43].

Since the differing results of studies on breast cancer in *BRCA1* carriers undergoing prophylactic salpingo-oophorectomy came from multi-center, multi-national trials performed in various countries: USA, Canada, Netherlands, France, Norway, Spain, Australia and New Zeeland, we believe that it is necessary to examine molecular background in all populations, particularly for such a common malignancy as breast cancer.

There were few publications on the influence of breast cancer diagnosis among *BRCA1* gene mutation carriers before prophylactic surgery and the incidence of peritoneal cancer in the postoperative period. There are also few publications regarding the influence of the time from diagnosis of breast cancer to prophylactic surgery and the diagnosis of peritoneal cancer in the postoperative period.

In our material the diagnosis of breast cancer before prophylactic surgery was associated with an almost three-fold increase in the risk of peritoneal cancer. However, that correlation was not statistically significant. Domchek et al. acquired an opposite result. Peritoneal cancer was found less often in patients with *BRCA1* mutation and breast cancer diagnosis before surgery than in patients without breast cancer prior to surgery – 1.18 % vs. 1.75 %. Researchers demonstrated also that prophylactic salpingo-oophorectomy decreased the risk of primary peritoneal cancer among patients without previous diagnosis of breast cancer by 70 % and in patients diagnosed with breast cancer prior to prophylactic surgery by 85 % [22].

In our opinion, dissimilarities in our conclusions with regard to the risk of development of peritoneal cancer among patients without the diagnosis of breast cancer prior to prophylactic surgery may ensue from different numbers of diagnosed peritoneal cancer cases – 2 vs. 10 patients in a study by Domchek et al. Moreover, in our study both patients diagnosed with primary peritoneal cancer, who had been treated for breast cancer prior to prophylactic surgery, decided to have the salpingo-oophorectomy performed after the age of 50. Therefore, surgery might no longer serve a protective role in development of peritoneal cancer postoperatively.

Continuing the subject of the influence of breast cancer diagnosis before prophylactic surgery, among six analyzed features we found only the risk of death to be statistically significant. Patient survival after prophylactic surgeries will be the subject of another publication.

The topic of the influence of *BRCA1* gene mutation on examined characteristics is highly problematic to discuss. Although the type of mutation most commonly identified in Polish population - *5382insC –* is also found among Jewish people, Ashkenazi Jews in particular [46], available literature lacks the data for discussion.

## Conclusions

Based on the analysis of gathered material and existing literature we found that in our data the incidence of primary peritoneal cancer after prophylactic surgery is similar to that reported in international literature. What differs our material from reports in international literature is the occurrence of primary breast cancer – it was significantly less frequent. Another conclusion is that it cannot be excluded that the risk of breast cancer development among constitutional *BRCA1* gene mutation carriers subjected to prophylactic surgery varies in a population. The next goal in research should be to examine molecular background with regard to the risk of malignancy in every population. It is also important that carriers of the *BRCA1* gene mutation diagnosed with breast cancer should be subjected to prophylactic surgery within less than five years from the diagnosis of breast cancer, as the risk of peritoneal cancer increases significantly after that period.

### Consent

Written informed consent was obtained from the patient for the publication of this report and any accompanying images.
